# Connecting the nanoseed defect structure and crystallinity with resulting nanoparticle products†

**DOI:** 10.1039/d5na00418g

**Published:** 2025-05-16

**Authors:** Debashree Roy, Helen C. Larson, Brandi M. Cossairt, Liane M. Moreau

**Affiliations:** a Department of Chemistry, Washington State University Pullman WA 99164 USA liane.moreau@wsu.edu; b Department of Chemistry, University of Washington Seattle WA 98195 USA

## Abstract

Anisotropic gold (Au) nanocrystals (NCs) represent an interesting class of materials due to their shape and size dependent tunable optical properties as well as facet dependent catalytic and photocatalytic properties. The morphology of anisotropic Au NCs synthesized *via* the versatile seed-mediated synthesis is considered to be heavily dependent on the crystallinity and defect structure associated with the initial seed, modified in conjunction with surfactants and/or other shape-directing agents. The seeds can be considered as templates having well defined internal structure and crystal facets on which further atom deposition takes place *via* heterogeneous nucleation. While defect-structure directed morphological control has been established, the correlation of the seed crystal facets with the final morphology of Au NCs is rarely emphasized. In this study, we draw direct parallels between the crystal structure of the seed and the final morphology of Au NCs. We investigate this area by starting with seeds that have the same dominant crystal structure {111} but with four different morphologies and defect structures. Surprisingly, all the structures led to similar stellated NC products. Our findings open new avenues to evaluate NCs synthesized with seeds containing other crystal facets and exercise morphological control over Au NCs.

## Abstract 2

4 Au NC seeds bound by (111) planes but having different morphologies and defect structures were overgrown under identical conditions. Similar branched NCs resulted in all cases, emphasizing the critical role of the (111) plane itself.

## Graphical abstract

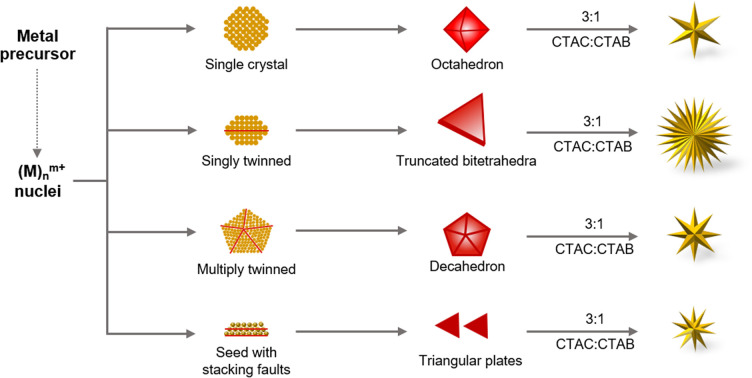

## Introduction

The interesting optical, electronic, and catalytic properties displayed by gold (Au) at the nanoscale, compared to its bulk counterpart, arise due to varying nanocrystal (NC) size and shape.^1–3^ The seed-mediated growth strategy is by far the most commonly employed synthetic route to achieve reproducible and tunable morphological control of anisotropic Au NCs in high yield.^4,5^ This high degree of shape control coupled with facile shape tunability arises due to separation of the initial nucleation of the atoms to generate the “seed” from the subsequent overgrowth of these seeds.^5^ Usually, the seed defect structure (or the internal structure) is preserved in the resultant Au nanostructures wherein single crystal seeds give rise to octahedrons, cuboctahedrons, cubes, single crystalline nanorods (NRs), or rectangular bars.^6–8^ Singly twinned seeds will more likely result in right bipyramid morphology, and multiply twinned seeds produce decahedrons, icosahedrons or pentatwinned NRs.^6–8^ In contrast, seeds exhibiting stacking faults grow into triangular or hexagonal plates.^9–11^ Additionally, NC growth pathways can also be completely rerouted to result in shapes that differ from the ones outlined above in the presence of strong-binding surface capping agents working in tandem with the seed structure.^12–24^

This correlation of seed morphology with metal NC formation has been extensively studied by Skrabalak *et al.*, with particular emphasis on seed structure, seed defects, and symmetry transfer in the resultant bimetallic NCs.^25–28^ Specifically, their study on bimetallic NCs synthesized *via* seed-mediated co-reduction has shown how the seed composition is an important parameter in controlling the final bimetallic NC morphology.^26^ Different products were observed when single crystalline Au seeds were used *vs.* when Pd seeds were used. Thus, the guidelines that can be inferred from the results for bimetallic nanostructures may not necessarily hold for monometallic systems, since the surface characteristics and interatomic bonding energies will be different.^29–31^

In contrast to the bimetallic systems referenced above, limited knowledge is available about the intrinsic relationship between the final morphology of monometallic NCs and the crystal facets of the starting seeds. Therefore, this work focuses on this relationship as the subject of the current study. Seed crystal facets refer to the crystal planes on the surface of the seed.^32^ Several instances exist where different starting seeds bound by different crystal facets give rise to similar morphology, albeit under different synthetic conditions.^20,33–53^ The most common example is the formation of single crystalline Au NRs from single crystalline cuboctahedral seeds and pentatwinned Au NRs from decahedral seeds.^54,55^ Single crystalline Au cuboctahedra which are typically used to synthesize single crystalline Au NRs are enclosed with a mix of low index crystal facets {100} and {111} as evidenced recently *via* high resolution transmission electron microscopy (HRTEM) images.^56^ On the other hand, decahedral seeds used to synthesize pentatwinned Au NRs have an internal defect in the form of a pentagonal twin axis and are bound by {111} crystal planes.^55,56^ Additionally, formation of a universal seed to result in the formation of different anisotropic Au NCs under different synthetic conditions has also been established.^57^

Despite the significance of surface crystalline structures (or crystal facets) in the growth and morphological evolution of Au NCs, no research to the best of our knowledge has been put forward to establish their correlation with the final Au NC morphology. We limit the goal of the present study to correlating the seed crystal facet with the resulting NC morphology in monometallic Au NC syntheses. The growth mechanism is the subject of a different study and will be considered elsewhere. To this end, we observe that despite different seed shapes and defect structures, similar final morphologies were obtained when Au NCs bound by the same crystal facets but with different morphologies were chosen as the starting seeds. Accordingly, NCs bound exclusively by {111} crystal facets such as octahedra, truncated bitetrahedra (TBT), decahedra, and triangular plates were included in the current study as seeds. All of these different NCs are exclusively bound by {111} crystal facets and also exhibit different defect structures (single crystalline, singly twinned, multiply twinned, and having stacking faults, respectively).

Here, we demonstrate for the first time the overgrowth of seeds in monometallic Au systems, with diverse internal defect structures bound by the same surface crystal facets. Under identical reaction conditions in conjunction with the quaternary ammonium halide surfactants, these varied structures all generated stellated (branched) Au NCs. Furthermore, we show that seeds bound by crystal facets besides {111} planes are unable to result in stellated Au NCs. This reveals a finding that gives precedence to the surface facets of the seed rather than only the seed defect structure and the dominance of the former over the latter.

The purpose of the current study is not to synthesize superior stellated Au NCs, hence, lesser emphasis has been laid on characterizing the stellated Au NCs. Our findings are nonetheless crucial to understanding the importance of surface facets of the starting seed in directing the resultant Au NC growth. Furthermore, our results suggest that the exact seed morphology, as long as there are {111} planes, may not have a large effect on the resulting product. It is likely that these results can be translated to the synthesis of other anisotropic Au NCs with seeds bound by different surface facets.

## Methods

### Chemicals

Silver(i) nitrate, l-ascorbic acid, tannic acid, gold(iii) chloride trihydrate, cetyltrimethylammonium chloride, benzyldimethylhexadecylammonium chloride, and sodium borohydride were purchased from Sigma Aldrich. Sodium hydroxide (ACS-grade) was bought from Macron, and cetyltrimethylammonium bromide was purchased from Bioworld. Sodium iodide and citric acid were purchased from J. T. Baker Inc. All chemicals were used as received without additional purification. All H_2_O used to make solutions was UltraPure Type 1 Deionized (DI) Water (ChemWorld). All glassware was washed with aqua regia prior to use and all solutions were used fresh.

### Synthesis of Au octahedral NCs (with citric acid)

A new synthetic protocol was developed to synthesize Au octahedral NCs using citric acid. Briefly, to a solution containing 3.75 mL DI water, 10 mL 0.5 mM HAuCl_4_, 5 mL 0.2 M CTAC and 1 mL 0.1 M citric acid, 0.25 mL 25 mM freshly prepared NaBH_4_ was added under vigorous stirring at room temperature. The mixture turned from light yellow to a brownish tint upon addition of NaBH_4_. After 2 minutes, the solution was sealed and heated at 80 °C for 90 minutes under gentle stirring leading to a gradual change of color from brown to pink. The Au octahedral NCs were used in overgrowth procedures without any further purification.

### Synthesis of Au octahedral NCs (without citric acid)

Au octahedral NCs were synthesized by modifying a previously reported protocol.^58^ Briefly, a seed solution was prepared using 25 μL 10 mM HAuCl_4_ and 1 mL 0.1 M CTAC, to which 60 μL of freshly prepared 0.1 M NaBH_4_ was added, and the mixed solution was stirred for 1 minute and left undisturbed for 2 hours.

For the growth solution, 5 mL DI water, 5 mL 0.2 M CTAC, 1.25 mL 4 mM HAuCl_4_, 2.5 μL 4 mM AgNO_3_, and 200 μL 0.1 M HCl were consecutively added. This was followed by the addition of 100 μL 0.1 M ascorbic acid. 100 μL of the seed diluted 100 times in 0.1 M CTAC was introduced to initiate the reaction. The resultant Au octahedral NCs were used in overgrowth procedures without further purification.

### Synthesis of Au TBT NCs

Au TBT NCs were synthesized following a previously reported protocol.^42^ Briefly, a seed solution was prepared using 25 μL 10 mM HAuCl_4_ and 1 mL 0.1 M CTAC, to which 60 μL of freshly prepared 0.1 M NaBH_4_ was added, and the mixed solution was stirred for 1 minute. The solution was then left undisturbed for 2 hours before being used to prepare larger seeds.

To prepare larger seeds, a solution containing 0.8 mL DI water, 160 μL 100 mM CTAC and 20 μL 10 mM HAuCl_4_ was prepared. To this solution, 4 μL 0.1 M ascorbic acid was added. This was followed by the addition of 10 μL seed as described in the previous paragraph, and the mixed solution was diluted 10 times in 0.1 M CTAC.

The growth solution was prepared by adding 7.5 mL DI water, 2.5 mL 0.2 M CTAC, 0.5 mL HAuCl_4_, 75 μL 10 mM NaI and 100 μL 100 mM ascorbic acid. Then, 0.5 mL of the larger seed solution was introduced without any further dilution to initiate the reaction. The as-synthesized TBT Au NCs were used as seeds for further overgrowth.

### Synthesis of Au decahedral NCs

Au decahedral NCs were synthesized following a previously reported protocol.^59^ Briefly, to prepare the seed solution, 2.65 mL 25 wt% CTAC and 400 μL 25 mM HAuCl_4_ were mixed with 33 mL DI water and heated to 30 °C for 10 minutes. This was followed by the addition of 4 mL 50 mM sodium citrate, and the heating was continued for 30 minutes. 1 mL 25 mM freshly prepared NaBH_4_ was added under fast stirring. The bottle was sealed and placed in an oven with temperature maintained at 40 °C for 5 days. For the growth solution, 500 μL 25 mM HAuCl_4_ was added to 25 mL 100 mM BDAC, followed by the addition of 188 μL 100 mM ascorbic acid. Under fast stirring, 50 mL of the seed solution was introduced, and the reaction was left to stir slowly for 30 minutes at 30 °C. The resultant Au decahedral NCs were used for overgrowth procedures without further purification.

### Synthesis of Au triangular plates

A seedless method previously reported was followed to prepare the Au triangular plates.^60^ Briefly, to 10 mL 50 mM CTAB solution, 275 μL 10 mM HAuCl_4_ and 10 mL 50 mM NaI were added in quick succession. This was followed by the addition of 55 μL 100 mM ascorbic acid and 8 μL 25 mM NaBH_4_, which resulted in a clear purple solution. The triangular plates were used for overgrowth procedures without further purification.

### Synthesis of single crystalline Au small seeds

A previously reported synthetic protocol was modified to synthesize Au cuboctahedra. Briefly, 500 μL ultrapure DI water was added to 625 μL 0.2 M CTAB, followed by the addition of 78 μL 4 mM HAuCl_4_. 45 μL 20 mM freshly prepared NaBH_4_ was added to the solution to initiate the reaction under stirring.^54^ The reaction was stirred for 5 minutes and left undisturbed for 10 minutes before being used in the overgrowth procedure without any further purification.

### Synthesis of Au nanorods

Au cuboctahedra synthesized in the previous section were used as seeds to synthesize Au NRs.^61^ The growth solution was prepared by adding 630 μL ultrapure DI water, 1 mL 0.2 M CTAB, 250 μL 4 mM HAuCl_4_, and 50 μL 4 mM AgNO_3_, followed by the addition of 20 μL 100 mM NaOH and 10 μL tannic acid. The reaction was initiated by the addition of 50 μL of cuboctahedral seeds. The nanorods were subsequently used as seeds for overgrowth control experiments.

### Synthesis of stellated Au nanocrystals

To synthesize Au nanostructures, a growth solution was prepared by adding 702.5 μL ultrapure DI water, 750 μL 0.2 M CTAC, and 250 μL 0.2 M CTAB (to make the volume up to 1 mL), 250 μL 4 mM HAuCl_4_, 10 μL 4 mM AgNO_3_, and 20 μL 100 mM NaOH. This was followed by the addition of 10 μL 100 mM tannic acid, which resulted in a color change of the solution from yellow to pale tan. Following the color change, 7.5 μL of the different seed solutions outlined were added to the growth solutions to initiate the reaction, and the mixed solutions were left undisturbed overnight. The resultant Au NCs were centrifuged thrice at 8663×*g* RCF for 5 minutes for purification and to enable further characterization.

## Characterization methods

### Transmission electron microscopy (TEM)

TEM images were taken using an FEI Tecnai G2 20 Twin TEM equipped with a 200 kV LaB_6_ electron source. Images were collected in bright field mode using a 4K Eagle camera. Samples were prepared by drop-casting 10 μL of centrifuged samples onto a carbon film 400 mesh Cu TEM grid and air dried.

### High-resolution transmission electron microscopy (HRTEM)

HRTEM imaging was performed on a FEI Tecnai G2 F20 SuperTwin microscope operated at 200 kV using the bright field mode. Samples were prepared by drop-casting 10 μL of centrifuged samples onto a carbon film 400 mesh Cu TEM grid and air dried. ImageJ was used to generate Fast Fourier Transforms (FFTs) of full or partial images for analysis. The contrast and brightness of the FFTs were adjusted using ImageJ.

Selected area electron diffraction (SAED) was performed on a FEI Tecnai G2 20 Twin microscope operated at 200 kV. The resolution limit for SAED on this microscope set up was approximately 0.5 microns.

### Scanning electron microscopy (SEM)

SEM images were collected using a FEI Apreo VolumeScope SEM. The samples for SEM imaging were prepared by dispersing the sample in DI water and drop-casting 10 μL of the respective sample dispersions on ultra-flat silicon wafers and allowed to dry overnight in a desiccator.

## Results and discussion

**Scheme 1:**
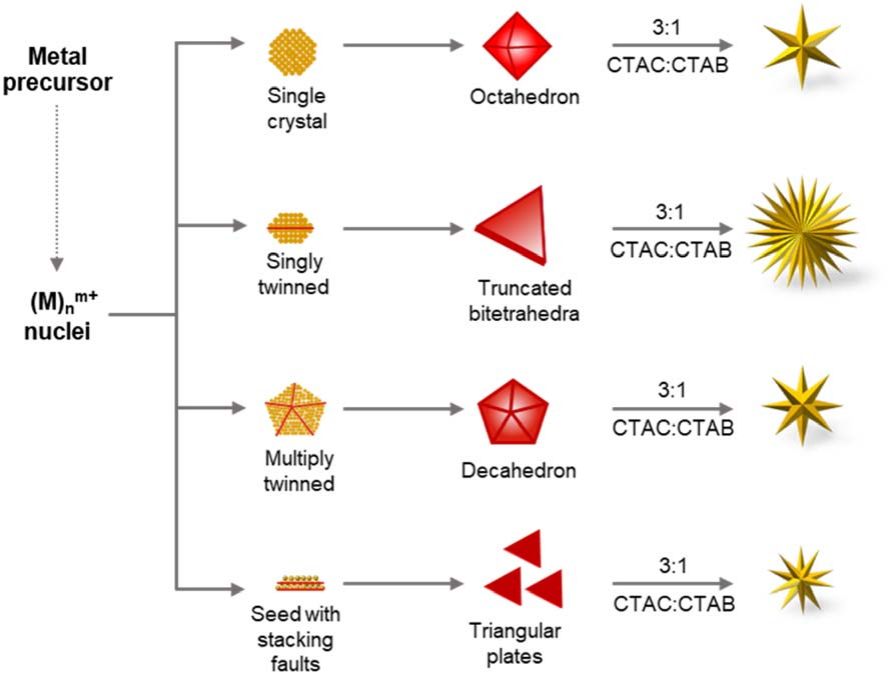
Schematic representation of the growth of our synthesized Au seeds with different defect structures and morphologies but the same {111} surface crystal facets. These Au NCs were subjected to overgrowth under identical synthetic conditions giving rise to stellated Au NCs.

**Fig. 1:**
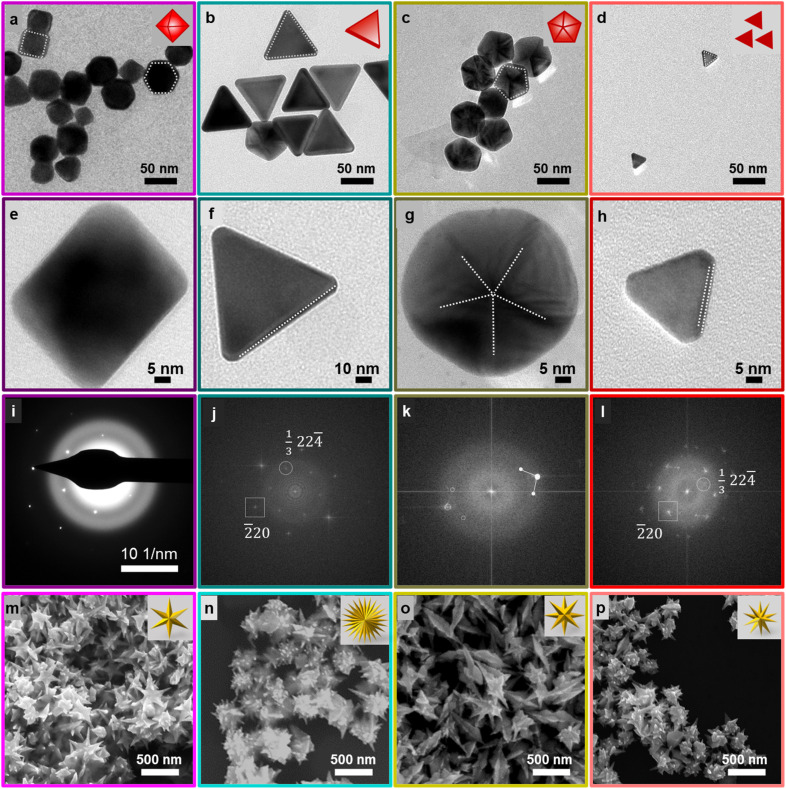
Low magnification TEM images of the different Au NCs used as seeds indicate their successful syntheses and shape purity: (a–d) Au octahedra, Au TBT, Au decahedra, and Au triangular plates, respectively. (e–h) Zoomed-in TEM images of four different seeds with distinct defect structures: single crystalline Au octahedra, singly twinned Au TBT NCs, pentatwinned Au decahedra, and triangular plates with stacking faults, respectively. (i–l) Corresponding electron diffraction obtained from Au octahedra and FFT patterns obtained from Au TBT, Au decahedra, and Au triangular plates. (m–p) SEM images of stellated Au NCs overgrown from respective seeds under identical synthetic conditions.

Four Au seeds differing in their defect structure but exhibiting the same {111} planes at their surfaces were chosen for this study. Based on the number of planar defects present in a seed, NCs can be classified into four major categories, specifically single crystal seeds having no planar defects, singly twinned seeds exhibiting a single twin boundary, multiply twinned seeds showing more than one planar defect, and seeds lined with stacking faults.^5^ Starting seeds chosen in this study are representative of each category. Each is bound exclusively by {111} planes: (1) single crystalline Au octahedral seeds, (2) singly twinned truncated bitetrahedra (TBT), (3) pentatwinned Au decahedra, and (4) Au triangular plates having stacking faults (Scheme 1). These NCs were synthesized following previously reported synthetic protocols.^42,58–60^ TEM images of representative seed particles confirmed that they were synthesized successfully (Fig. 1a–d). HRTEM images confirmed the defect structures associated with each of the respective seeds: no crystal defects (Au octahedra), single twinning (Au TBT), pentatwinning (Au decahedra), and stacking faults (Au triangular plates) (Fig. 1e–h and S1–S3†). The corresponding electron diffraction and FFT patterns (Fig. 1i–l) coupled with the HRTEM micrographs (Fig. S1–S3†) exhibiting the lattice fringes allow for the determination of inter-planar spacing *d*. Single particle FFT patterns confirm that the respective NCs are covered with {111} surfaces (Fig. S1–S3†).^6,7,62–65^ The four different types of seeds were subjected to overgrowth in the presence of binary surfactants commonly used in the synthesis of anisotropic Au NCs (cetyltrimethylammonium bromide (CTAB) and cetyltrimethylammonium chloride (CTAC)). All NCs were synthesized under identical growth conditions for direct comparison of products. Fig. 1m–p reveal the Au NC products formed from each of the four seeds. Interestingly, all four seeds created stellated structures, albeit with varying degrees of branching. The formation of stellated Au NCs from twinned seeds is not uncommon in the literature^66–68^ and often leverages on defects present in the starting seeds, with the growth of branches at the defect junctions often associated with high surface energy to minimize the overall NC energy. In fact, overgrowth of Au triangular plates to result in the formation of stellated Au NCs has been observed to have a direct correlation with the plate size and the number of branches of the resultant Au NCs.^27^ While overgrowth of Au triangular plates (edge lengths: 35 ± 5 nm) resulted in the formation of a two-branched structure, to achieve multi-branched structure, larger Au triangular plates (edge lengths: 350 ± 21 nm) were used as seeds.^27^ The triangular morphology is maintained in both cases. In contrast, when Au triangular plates (edge lengths: 21 ± 3 nm) were subjected to overgrowth in the present study, it led to the formation of highly branched nanostructures without the triangular morphology being preserved. This clearly indicates a different growth mechanism at play, unlike the overgrowth from the top and bottom of the nanoplates reported previously.^27^ The conditions leading to the formation of the stellated structures from reported single crystalline seeds were also found to be different.^69,70^ While, in one instance the overgrowth of the Au octahedra at the six vertices resulted in the formation of a branched structure with a controllable number of arms and tunable arm length,^69^ in the other report single crystalline seeds transformed into twinned seeds with {111} facets due to the low energy barrier associated with twinning and angle strain.^70^ This was aided by the presence of the high ascorbic acid concentration used as the reductant,^70^ contrary to the present study. On the other hand, single crystalline seeds (octahedra) have given rise to stellated NCs *via* overgrowth at the tips or branching perpendicular to the faces of the NCs.^26^ Importantly, our findings do not conform to the mechanisms reported in the literature^26,27,66–71^ to explain the formation of the stellated Au NCs. To the best of our knowledge, we report for the first time the formation of the same final stellated structures from seeds enclosed with identical crystal facets {111} but exhibiting different defect structures under otherwise identical synthetic conditions.

This further emphasizes the dominance of the surface facets over the seed defect structure in the current study. Single crystalline seeds used in the growth of single crystalline Au NRs were also subjected to overgrowth under identical reaction conditions. These single crystalline seeds have been studied extensively with the help of advanced microscopic techniques. The crystal structure was revealed to be a mixture of {111} and {100} planes without causing electron-beam damage to their structure.^56^ The overgrown products of the single crystalline Au seeds resemble Au NR morphology, bereft of stellation of any kind (Fig. S4a†). In the reported study^70^ where single crystalline seeds develop defects to lower the overall surface energy, structural perturbations in turn facilitate the formation of stellated Au NCs. In contrast, the ultrasmall single crystalline Au seeds employed as a control in the present study, despite having similar facet energies and being more conducive to structural perturbations,^64,72^ neither develop defects nor lead to stellated morphologies. Furthermore, single crystalline Au NRs subjected to overgrowth under identical synthetic conditions did not yield stellated Au NCs either (Fig. S4b†). These Au NRs were synthesized *via* a highly reproducible reported synthetic protocol,^54^ and as such are considered to be enclosed by {111} and {110} planes at the tip and higher index {520} lateral facets.^73–75^ A previous report^26^ on the formation of stellated morphology from Au NRs and the absence of stellated morphology formation from Au NRs in the current study not only underline the importance of the seed crystal facet but also a growth mechanism that gives precedence to the seed crystal facet. This confirms the assumption that seeds bound exclusively by {111} facets are crucial for the growth of stellated Au NCs under the synthetic conditions employed in the present study. This finding also suggests that the single crystalline Au octahedral seeds do not develop defects to generate the stellated Au NCs while affirming the critical role of the seed crystal structure over the seed defect structure in defining the final Au NC morphology.

It is well established in the Au NC literature that the presence of multiply twinned defects in the starting seeds can induce the formation of stellated Au NCs, and the presence of citric acid is known to promote multiply twinned Au seed particles.^76,77^ Since the Au octahedral NCs employed as seeds in the current study were synthesized using citric acid, it was important to rule out the possibility that the subsequent formation of stellated Au NCs was solely due to the presence of defects. Single crystalline octahedral NC structures still resulted in stellated products. To affirm that, control experiments were conducted where Au octahedral NCs synthesized following a citric acid free protocol where CTAC and ascorbic acid were employed instead.^58^ These Au octahedral NCs were subjected to overgrowth under otherwise identical synthetic conditions. These citric acid free NCs also resulted in the formation of a stellated morphology (Fig. S5†), providing additional credibility to our hypothesis of the seed crystal {111} facet dominating over the seed defect structure.

The hypothesis for this work is developed from the universally accepted understanding that surfactants work by modifying the surface energy of specific facets.^78^ Thus, the presence of different crystal facets on the seed will complicate the growth process due to differences in surface energies associated with different crystal facet growth rates. This is observed in the case where ultrasmall single crystalline Au NCs and Au NRs were used as seeds. On the other hand, we employed starting seeds that are bound by the same crystal facet irrespective of the seed defect structure or morphology. By virtue of the same surface energy associated with the said crystal facets, the growth rates will be the same under identical reaction conditions. This will direct the reactions to the same outcome. In other words, we observe that since the starting seeds were bound by the same crystalline facet (in this case {111}), the crystal defect structure and morphology notwithstanding, stellated structures are observed in all cases.

## Conclusions

In conclusion, we have definitively demonstrated that Au monometallic seeds with different defect structures but with {111} facets can be modulated to result in similar stellated NCs. The formation of stellated Au NCs under vastly different defect structures indicates that the crystal surface faceting plays a more pivotal role than has been previously considered. Importantly, our findings lead to a significant new understanding of anisotropic noble metal syntheses at the nanoscale and afford the opportunity to revisit established synthetic protocols with a new perspective.

## Abbreviations

NCs: nanocrystals
NR: nanorod
CTAB: cetyltrimethylammonium bromide
CTAC: cetyltrimethylammonium chloride
BDAC: benzyldimethylhexadecylammonium chloride
TBT: truncated bitetrahedra
TEM: transmission electron microscopy
SAED: selected area electron diffraction
SEM: scanning electron microscopy
FFT: fast Fourier transform

## Author contributions

The project was conceptualized by D. Roy, who also synthesized all particles and performed microscopy measurements. The HRTEM images and FFT analyses were carried out by H. C. Larson and B. M. Cossairt. The manuscript was written by D. Roy and L. M. Moreau. All authors have read through the manuscript and given approval to the final version of the manuscript.

## Conflicts of interest

There are no conflicts to declare.

## Supplementary Material

NA-007-D5NA00418G-s001

## Data Availability

Additional TEM, HRTEM, and SEM images along with FFT data supporting this manuscript have been included as part of the ESI.†
