# Supplementary material for: Connecting the nanoseed defect structure and crystallinity with resulting nanoparticle products
Source: Nanoscale Adv. 2025 May 16;7(14):4412–8. doi: 10.1039/d5na00418g (PMC12146894; doi:10.1039/d5na00418g)
Supplement: NA-007-D5NA00418G-s001 [file NA-007-D5NA00418G-s001.pdf]

Supplementary Information (SI) for Nanoscale Advances.
This journal is © The Royal Society of Chemistry 2025

Electronic Supplementary Information for

Connecting Nanoseed Defect Structure and Crystallinity with Resulting Nanoparticle Products

Debashree Roy,[a] Helen C. Larson,[b] Brandi M. Cossairt,[b] and Liane M. Moreau,[a]*

[a]Department of Chemistry, Washington State University, Pullman, WA 99164

[b]Department of Chemistry, University of Washington, Seattle, WA 98195

AUTHOR INFORMATION

Corresponding Author

*Email – liane.moreau@wsu.edu

Table of Contents:

Figure S1. HRTEM images of Au TBT NCs and the corresponding FFT patterns....................**S3**

Figure S2. HRTEM image of pentatwinned NC and the corresponding FFT patterns.............**S4**

Figure S3. HRTEM image of Au TP NCs and the corresponding FFT patterns...........................**S5**

Figure S4 SEM images of Au NCs synthesized from Au cuboctahedra and Au NRs................**S6**

Figure S5. SEM images of stellated Au NCs synthesized from Au octahedra..........................**S7**

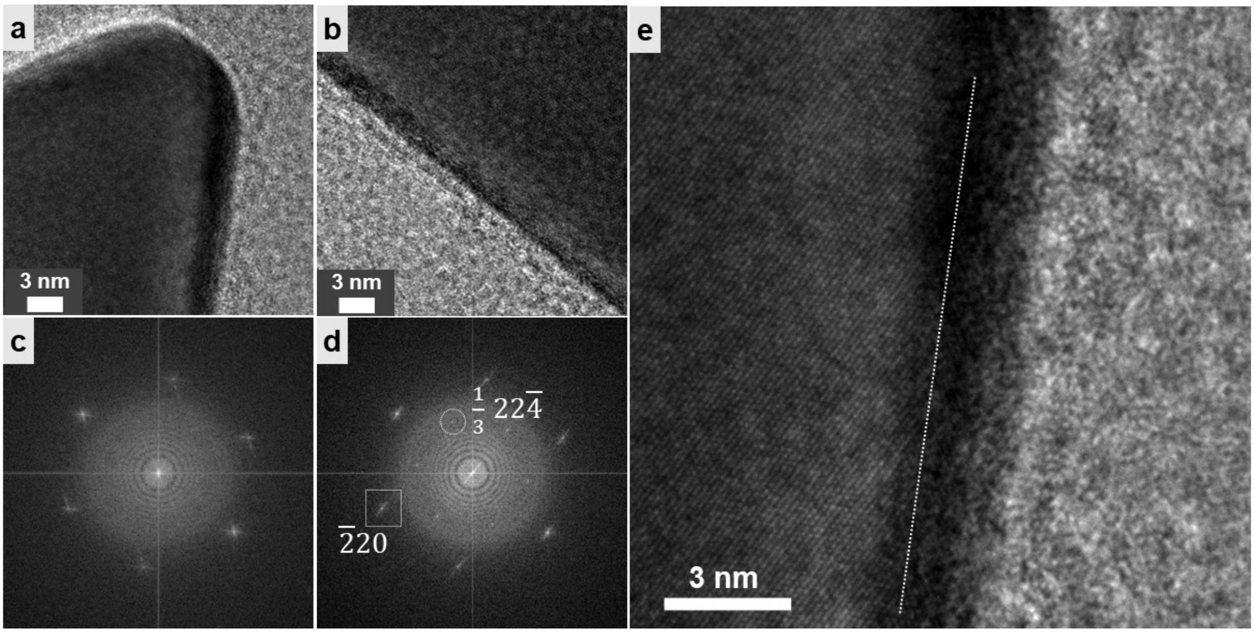

Figure S1. HRTEM images (a-b) obtained from different regions of a TBT Au NC and the FFT patterns (c-d) taken perpendicular to the triangular face of Au TBT NC is characteristic of the {111} orientation and shows the presence of the intense (220) and forbidden 1/3 (224) spots. The intense (220) spots correspond to the lattice spacing of 1.4 Å while the weak forbidden 1/3 (224) spots correspond to the lattice spacing of 2.5 Å. Additional spots observed in (d) surrounding the (220) spots arise due to the presence of a twin plane. (e) Magnified HRTEM image indicates the presence of a twin plan.

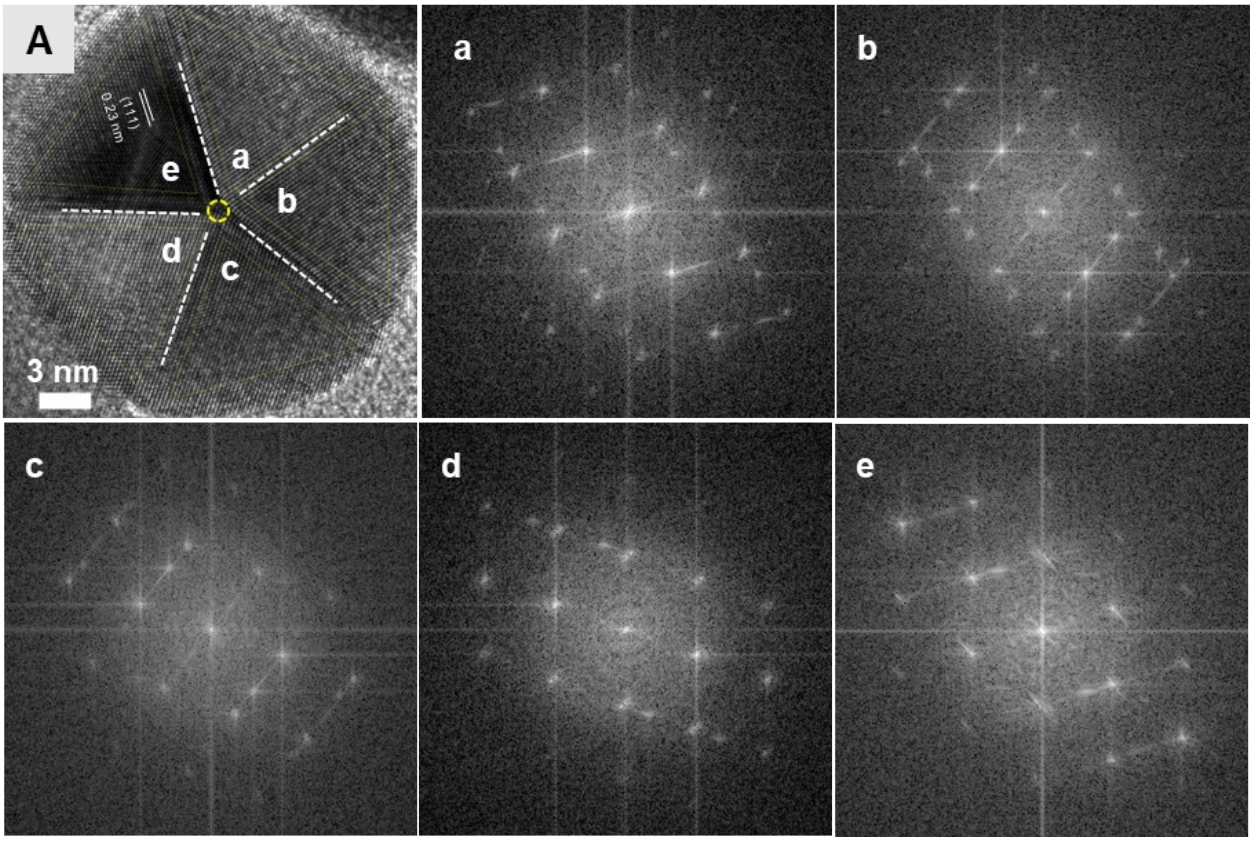

Figure S2. (A) HRTEM image of a pentatwinned NC exhibiting a five-fold twinning axis (yellow dashed circle) along the twin boundaries in white dashed lines. The five tetrahedral units labelled from a-e are highlighted in triangular patterns. (a-e) Corresponding FFT patterns obtained from the five different tetrahedral units.

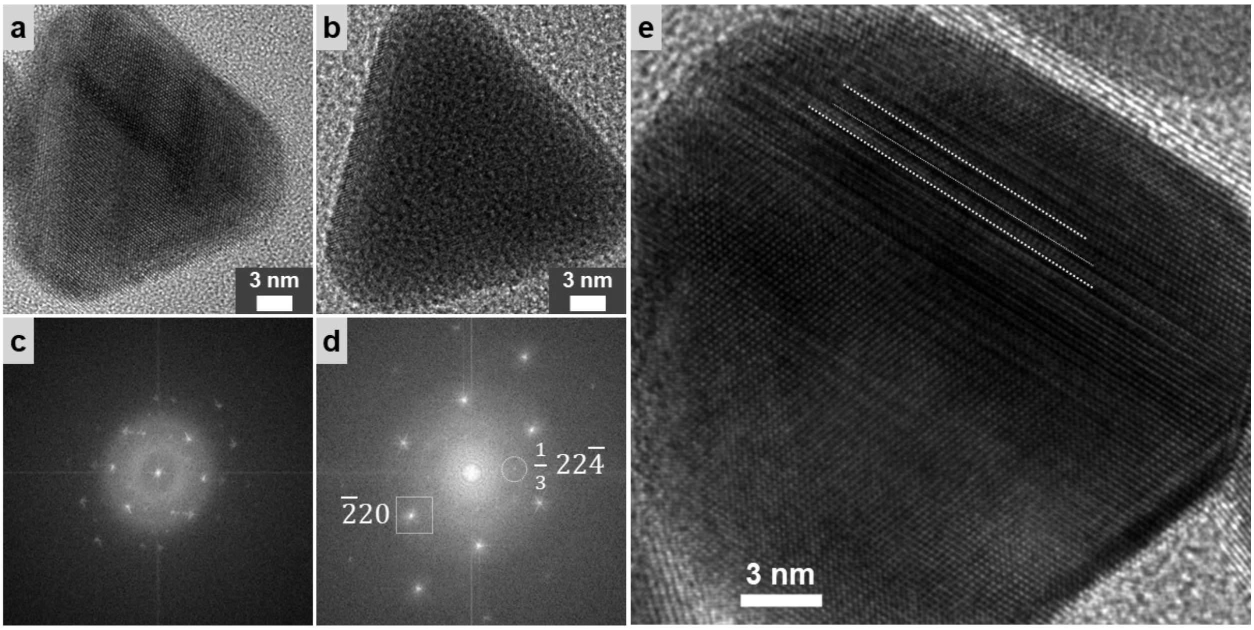

Figure S3. HRTEM images (a-b) obtained from different Au triangular plate NC and the corresponding FFT patterns (c-d) from the respective NCs taken perpendicular to the main face. This shows a <111>-type pattern as well as the presence of (220) and forbidden 1/3 (224) spots. Additional spots observed in (c) arise due to the presence of stacking faults. (e) Magnified HRTEM image of Au triangular plate NC indicates the presence of stacking faults highlighted in white.

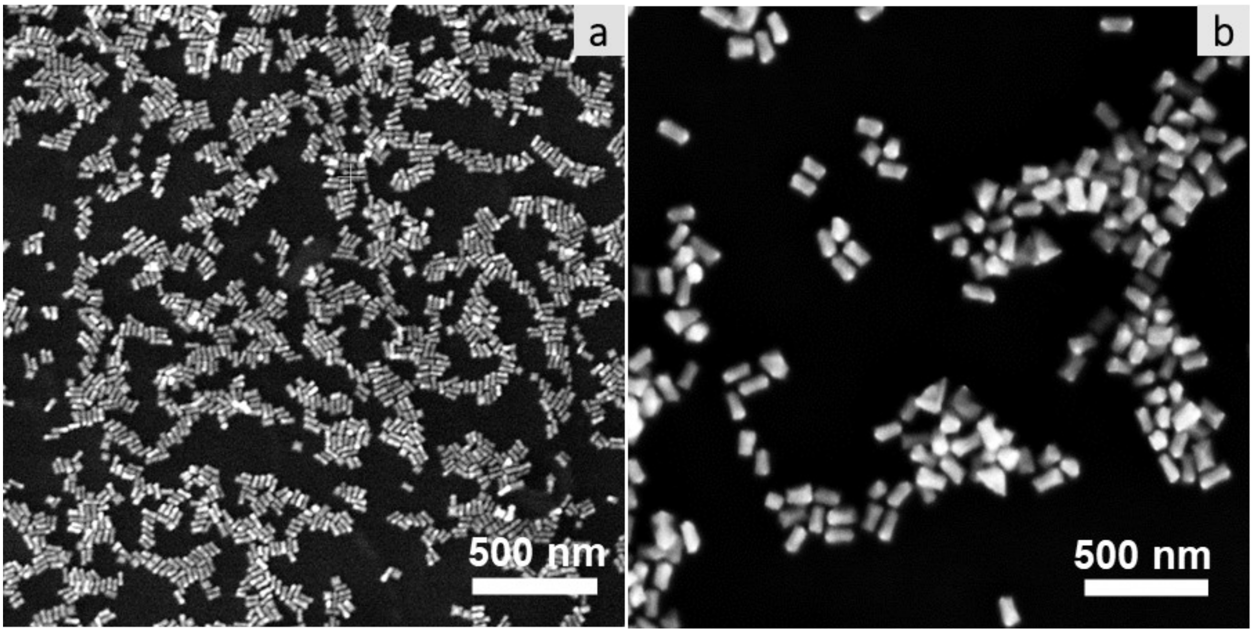

Figure S4. SEM images of Au NCs synthesized from (a) single-crystalline small Au seeds and (b) single-crystalline Au nanorods show that the final Au NCs are bereft of any stellation.

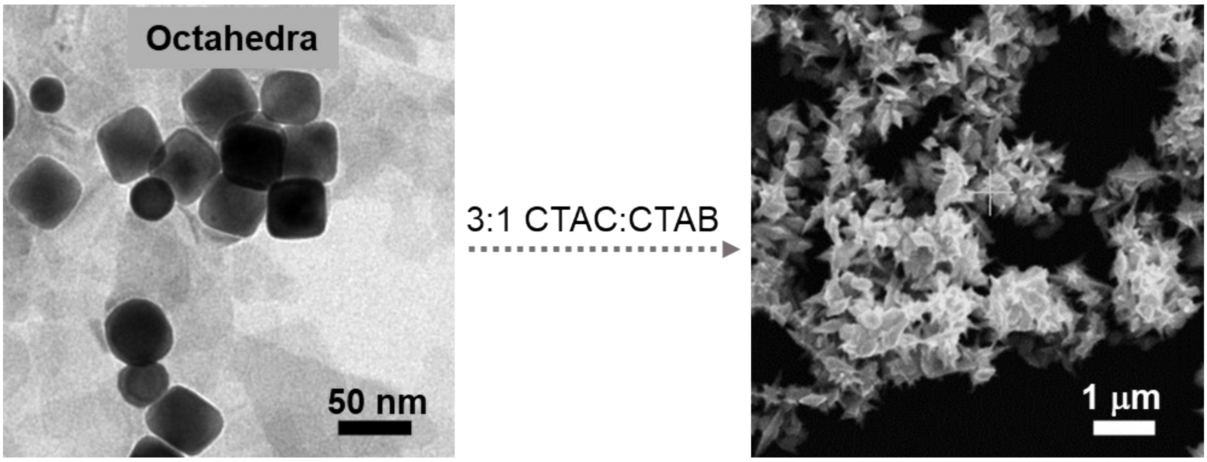

Figure S5. TEM image of Au octahedral NCs synthesized under citric acid free conditions. Overgrowth in the presence of a binary surfactant mixture of CTAC and CTAB surprisingly resulted in stellated Au NCs as imaged using SEM given the lack of citric acid.
